# Aldh2 deficiency plays a dual role in lung tumorigenesis and tumor progression

**DOI:** 10.1016/j.gendis.2023.04.030

**Published:** 2023-06-24

**Authors:** Hongjia Zhang, Xueqian Sun, Zhanming Li, Tingting Liu, Fang Zhang, Xinyu Meng, Kaimi Li, Jianhua Xu, Wei He, Bo Jing, Tong Wang, Na Ni, Beibei Sun, Feng Yao, Yadi Wu, Qi Wang, Jing Du, Eugene Y. Chin, Binhua P. Zhou, Ping Jiang, Lishun Wang, Jiong Deng

**Affiliations:** a Key Laboratory of Cell Differentiation and Apoptosis of Chinese Minister of Education, Department of Pathophysiology, Shanghai Jiao Tong University School of Medicine, Shanghai 200025, China; b Shanghai Key Laboratory for Tumor Microenvironment and Inflammation, Shanghai Jiao Tong University School of Medicine, Shanghai 200025, China; c Department of Pathology, Kunming Medical University, Kunming, Yunnan 650500, China; d Medical Research Center, Binzhou Medical University Hospital, Binzhou, Shandong 256600, China; e Center for Traditional Chinese Medicine and Gut Microbiota, Minhang Hospital, Fudan University, Shanghai 201100, China; f Department of Radiation Oncology, Yantai Affiliated Hospital of Binzhou Medical University, Yantai, Shandong 264100, China; g Department of Pathology, Molecular Pathology Research Center, Peking Union Medical College Hospital, Chinese Academy of Medical Sciences and Peking Union Medical College, Beijing 100730, China; h Department of Laboratory Medicine, Shanghai Pulmonary Hospital Affiliated Tongji University, Shanghai 200080, China; i Department of Physiology, School of Basic Medical Sciences, Weifang Medical University, Weifang, Shandong 261021, China; j Department of Thoracic Surgery, Shanghai Chest Hospital, Shanghai Jiao Tong University, Shanghai 200030, China; k Department of Molecular and Cellular Biochemistry, Markey Cancer Center, University of Kentucky College of Medicine, Lexington, KY 40506, USA; m Department of Respiratory Medicine, The Second Affiliated Hospital, Dalian Medical University, Dalian, Liaoning 116023, China; n Peninsular Cancer Center, Binzhou Medical University, Yantai, Shandong 264003, China

Alcohol consumption contributes to global mortality and cancer development. Acetaldehyde (ACE), the oxidized metabolite of alcohol, is highly reactive towards DNA, resulting in DNA adducts. ACE can be detoxified to acetate by acetaldehyde dehydrogenase type 2 (ALDH2). ALDH2 deficiency can lead to ACE accumulation and DNA damage.^1^ Thus, ALDH2 deficiency is considered pro-oncogenic.^2^ Interestingly, there are more than 540 million people carrying a polymorphism of the enzyme, ALDH2.2∗ a dominant-negative variant that has substantially reduced enzymatic activity. This suggests that ALDH2 deficiency is well tolerated in humans. Surprisingly, a recent epidemiological report shows that the risk of homozygotes of ALDH2.2∗ carriers for IARC alcohol-related cancer and lung cancer is significantly reduced, rather than increased, in men.^3^ This is contradictory and perplexing since ACE or reactive oxidative species (ROS) is pro-oncogenic. However, there is no explanation.

To investigate the roles of ALDH2 in cancer development, we analyzed ALDH2 mRNA in normal and tumor tissues of various cancers in the TIMER database. ALDH2 expression was low in tumor tissues compared to that in normal tissues for most cancers, including lung adenocarcinoma (LUAD) and lung squamous cell carcinoma (LUSC) (Fig. S1A). Moreover, ALDH2 was significantly reduced in tumor stage IV as compared to tumor stage I (*P* = 0.0172) (Fig. S1B). Consistently, The Cancer Genome Atlas (TCGA) data showed that the overall survival rate of LUAD patients with low ALDH2 was significantly shorter than those with high ALDH2 (*P* = 0.0042) (Fig. S1C). Thus, ALDH2 deficiency is associated with lung tumor progression.

Next, we examined the roles of Aldh2-deficiency in lung cancer cells. Mouse lung cancer cells S1601-shAldh2 were more aggressive *in vitro* than S1601-shNS (non-specific) in migration (Fig. 1A, B). Similarly, H1792-shALDH2 and A549-shALDH2 exhibited increased activities in colony forming and invasion compared to parental cells (Fig. S2A–F). Metabolically, ROS levels were higher in S1601-shAldh2 than in S1601-shNS cells, and ACE treatment further enhanced ROS levels (Fig. 1C; Fig. S3A). Consistently, S1601-shAldh2 cells produced more lung metastasis *in vivo* than S1601-shNS cells (Fig. 1D). Increased ROS (Fig. 1H; Fig. S3B) and migration (Fig. 1E–G; Fig. S3C, D) were also observed in A549-shALDH2 and H1792-shALDH2 cells, compared to their parental cells. Thus, ALDH2 deficiency promotes tumor progression in both mouse and human lung cancer cells.

**Figure 1:**
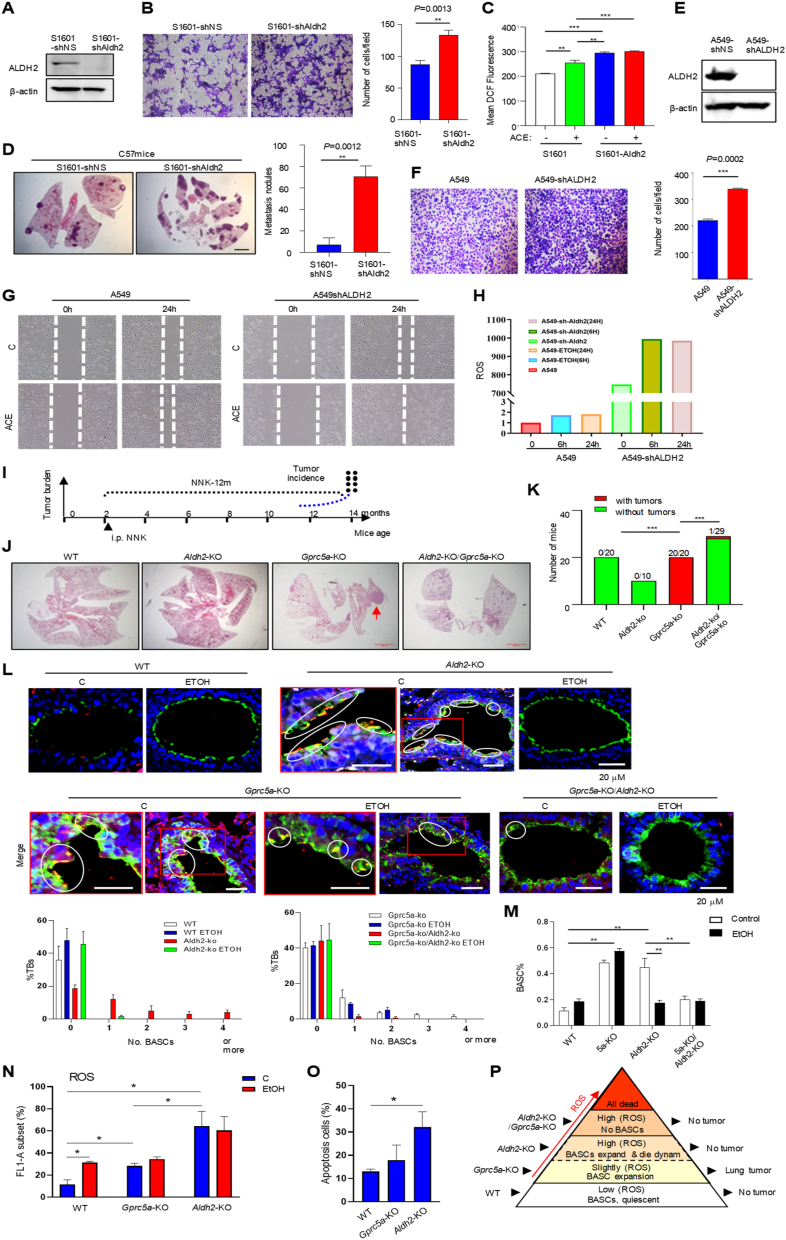
ALDH2 deficiency plays a dual role in lung tumor tumorigenesis and tumor progression. **(A)** Immunoblot of cell lysates from mouse lung cancer cells S1601-shNS and S1601-shAldh2. **(B)** Invasion of the cell lines *in vitro*. **(C)** Mean ROS by flow cytometry in mouse lung cancer cell line S1601 and S1601-shAldh2 treated with or without acetaldehyde (ACE). **(D)** Representative images of H&E staining of lung tissues from C57 mice intravenously injected with S1601-NS and S1601-shAldh2. Lung tissues were harvested three weeks after injection. Bar = 50 μm (E) or 200 μm (F). **(E)** Immunoblot of cell lysates from NSCLC cells A549-shNon-Specific (NS) and SA549-shALDH2. **(F)** Invasion of these cell lines *in vitro*. **(G)** The migration capacity of A549 and A549-shALDH2 cells that were treated with or without 1 mM ACE for up to 24 h, was analyzed by wound healing assays. **(H)** ROS levels in various lung cancer cell lines treated with or without ACE (1 mM) for 24 h by flow cytometry. **(I)** The schema of generation of *Gprc5a*-KO/*Aldh2*-KO mice. **(J)** Representative images of H&E staining of the lung tissues from WT, *Gprc5a*-KO, *Aldh2*-KO, and *Gprc5a*-KO/*Aldh2*-KO mice, 14 months after NNK stimulation. Bar = 100 μm. **(K)** The incidence of lung tumorigenesis in WT, *Gprc5a*-KO, *Aldh2*-KO, and *Gprc5a*-KO/*Aldh2*-KO mice. **(L)** Immunofluorescent (IF) staining for detecting cells at small and terminal bronchi (S/TB) that co-express SPA and CC10. The lung tissues were obtained from WT, *Gprc5a*-KO, *Aldh2*-KO, and *Gprc5a*-KO/*Aldh2*-KO mice, with or without ethanol treatment for 48 h. Bar graph indicates percentages of terminal bronchi with the indicated numbers of BASCs. Bar = 50 μm. **(M)** Analysis by flow cytometry of BASCs isolated from the lung of WT, *Gprc5a*-KO, *Aldh2*-KO, and *Gprc5a*-KO/*Aldh2*-KO following treatment with ethanol or saline. ∗∗*P* < 0.01; ∗∗∗*P* < 0.001. **(N)** ROS levels (FL1-A subset) in lung cells from WT, *Gprc5a*-KO, and *Aldh2*-KO mice treated with ethanol (EtOH) or without by flow cytometry. **(O)** Apoptotic cells (%) from WT, *Gprc5a*-KO, and *Aldh2*-KO mice by flow cytometry via Annexin V staining. **(P)** The proposed model of the roles of ROS in regulation of BASCs for initiation in lung tumorigenesis. Data are presented as the mean ± standard deviation. ∗*P* < 0.05; ∗∗*P* < 0.01; ∗∗∗*P* < 0.001.

Next, we explored the role of *Aldh2*-KO in lung tumorigenesis using the *Gprc5a*-KO mouse model. GPRC5A, a retinoic acid target, is preferentially expressed in lung tissue. *Gprc5a*-KO does not affect lung development but confers the susceptibility to lung tumorigenesis.^4^ Mice were intraperitoneally injected with tobacco carcinogen NNK, and lung tissues were harvested 12 months later (Fig. 1I). The results showed that: i) none of the 20 wild-type (WT) mice developed lung cancer (0%, 0/20); ii) none of the 10 *Aldh2*-KO mice developed lung cancer (0%, 0/10); ii) all the 20 *Gprc5a*-KO (5a-KO) mice developed lung cancer (100%, 20/20); and iv) only one of the 29 *Gprc5a*-KO/*Aldh2*-KO mice (1/29) developed lung cancer (3.3%) (Fig. 1J and K). We conclude that: i) *Aldh2*-KO does not confer the susceptibility to lung tumorigenesis; and ii) *Aldh2*-KO suppresses, rather than enhances, lung tumorigenesis in *Gprc5a*-KO mice. Of note, Aldh2 expression in lung tumors was indeed reduced compared to that in normal lung tissues in this model (Fig. S4A, B). This raises a question about why *Aldh2*-KO suppresses lung tumorigenesis while Aldh2 expression is repressed in lung tumors.

Tumor development is a process composed of three stages, initiation, promotion, and progression. Initiation is a process in which genetic driver mutations hit the cell of origin for carcinogenesis. Next, we examined bronchioalveolar stem cells (BASCs), localized in the bronchioalveolar duct junction (BADJ) in terminal bronchioalveoli (TBs). BASCs are the candidate cells of origin for lung cancer, in the *Kras*^*G12D*^ and *Gprc5a*-KO mouse models.^4^ Immunofluorescent (IF) staining showed that: i) there were few BASCs, SPA^+^ CC10^+^ (orange) cells, in WT mouse lungs (Fig. 1L; Fig. S5A); ii) BASCs were expanded in *Aldh2*-KO mouse lungs but eliminated when treated with ethanol (Fig. 1L; Fig. S5B); iii) BASCs were greatly expanded in *Gprc5a*-KO mouse lungs regardless of ethanol treatment (Fig. 1L; Fig. S5C); and iv) BASCs were eliminated in *Gprc5a*-KO/*Aldh2*-KO mouse lungs (Fig. 1L; Fig. S5D).

Flow cytometry analysis of BASCs, Sca-1^+^/CD34^+^/CD31^-^/CD45^-^ population, showed similar results: i) BASCs were increased in *Gprc5a*-KO and *Aldh2*-KO mouse lungs compared to WT ones; ii) BASCs were greatly reduced in *Gprc5a*-KO/*Aldh2*-KO mice; and iii) ETOH treatment eliminated BASCs in *Aldh2*-KO mice (Fig. 1M; Fig. S6). These suggest that ACE, due to *Aldh2*-KO background, is toxic to BASCs in mouse lungs.

Expanded BASCs are associated with susceptibility to lung tumorigenesis. However, why BASCs in *Aldh2*-KO mice were expanded but not susceptible to lung tumorigenesis? Intracellular ROS can be either stimulating or toxic to host cells depending on the concentration and the susceptibility of host cells. Next, we measured the ROS levels in mouse lung tissues and found that: i) ROS was low in WT mice; ii) ROS was slightly increased in ethanol-treated WT and *Gprc5a*-KO mouse lungs; and iii) surprisingly, ROS was drastically increased in *Aldh2*-KO mouse lungs, regardless of ethanol treatment (Fig. 1N; Fig. S7A, B). This suggests that *Aldh2*-KO generates a large amount of endogenous ROS in mouse lungs even without ethanol treatment.

To determine the impact of ROS in normal lungs, we isolated lung cells, digested and measured apoptotic cells in single-cell suspension by flow cytometry via annexin V staining, and found that: i) apoptotic cells were low in both WT and *Gprc5a*-KO mouse lungs; and ii) apoptotic cells were significantly increased in *Aldh2*-KO mouse lungs (Fig. 1O; Fig. S7C, D). We conclude that *Aldh2*-KO increased endogenous ROS, which is toxic and intolerant in normal lung tissues.

Cancer cells, via evolution, have gained the extra ability, such as an increased capacity of glutathione synthesis, to maintain redox homeostasis. ROS, such as ACE induced by ALDH2-deficiency, can be neutralized and tolerated in cancer cells. In fact, ACE treatment enhances the malignant features of lung cancer cells. Thus, ALDH2 deficiency is associated with malignant features and poor prognosis in patients with LUAD or LUSC.

Differently, initiation is induced in the cells of origin, or BASCs, in the lung.^5^ Normal cells are much more sensitive to ROS-mediated toxicity. This may explain why ALDH2-deficiency promotes the metastatic features in lung cancer cells,^1^^,^^2^ whereas *Aldh2*-KO is toxic to BASCs, especially with ethanol treatment. Previously, expanded BASCs (SPA^+^ CC10^+^) were proposed as the cells of origin for lung cancer in the transgenic *Kras*^*G12V*^ and the *Gprc5a*-KO mouse models.^5^ Here, lung tumorigenesis is strongly associated with the BASC status, expanded in *Gprc5a*-KO mice, eliminated in *Gprc5a*-KO*/Aldh2*-KO mice, and under great ROS-stress in *Aldh2*-KO mice. Although BASCs appear expanded in *Aldh2*-KO mouse lungs, they are in a dynamic process of expansion and apoptosis. In conclusion, ROS induced by ALDH2 deficiency can be tolerated in cancer cells, resulting in tumor progression; whereas ROS is intolerant in normal cells, resulting in the elimination of BASCs (Fig. 1P; Fig. S8). Of note, this assumption is strongly supported by a recent epidemiological investigation on an East Asian-specific loss-of-function variant of *ALDH2*, *ALDH2.2*∗ (rs671 G>A), an 11-year study of 150,722 adults. The risk (hazard risk, HR) of homozygotes of ALDH2.2∗ carriers, for IARC alcohol-related cancer, is significantly reduced in men (alcohol users) (HR, 0.69) but not in women (non-alcohol users) (HR, 1.12).^5^ From our experience, no gender difference in lung tumorigenesis was found in the applied mouse models (4). Nevertheless, our study provides a plausible explanation for *ALDH2.2*∗ paradox. We propose that ALDH2 deficiency plays a dual role in lung tumorigenesis and progression.

## Author contributions

JD and LW initiated and supervised the research. HZ, XS, ZL, TL, FZ, XM, K L, JX, WH, BJ, TW, NN, and BS performed most of the experiments and data analyses. FY, YW, JD, EYC, BPZ, and PJ provided crucial suggestions. HZ, XS, and ZL prepared the manuscript. All authors reviewed and approved the final version of this manuscript.

## Conflict of interests

The authors declare no conflict of interests.

## Funding

This work was supported by grants from the National Nature Science Foundation of China (No. 82172565, 81620108022, 81872245, 91129303, 91729302, 81572759, 31900441, 82003069, 82103571, 82002941, 82072570, 91129733, 81502702).

## Data availability

The data sets generated and/or analyzed during the current study are available from the corresponding authors upon reasonable request.
