# Supplementary material for: Aldh2 deficiency plays a dual role in lung tumorigenesis and tumor progression
Source: Genes Dis. 2023 Jun 24;11(3):100999. doi: 10.1016/j.gendis.2023.04.030 (PMC10825233; doi:10.1016/j.gendis.2023.04.030)
Supplement: Multimedia component 1 [file mmc1.docx]

**Appendix A.**

**Aldh2 deficiency plays a dual role in lung tumorigenesis and tumor progression**

**Materials and Methods**

**Mouse Models**

*Gprc5a*-knockout (KO) mice (*Gprc5a*^-/-^ mice) were generated in a mixed background of 129sv x C57BL/6 as described previously ^13,14^. Mice were maintained according to a protocol approved by Shanghai Jiao Tong University School of Medicine Animal Care and Use Committee [experimental animal use permission No: SYXK (Shanghai) 2008-0050] in the specific pathogen-free animal facility at the university. Eight-week-old wild-type (WT) and *Gprc5*a-KO mice were received 2 weekly intraperitoneal (i.p.) injections of 4-(N-Methyl-N-nitrosamino)-1-(3-pyridyl) -1-butanone (NNK) (100 mg/kg of body weight) (Midwest Research Institute, Kansas City, MO) dissolved in saline solution (0.9% NaCl) or saline alone (n=10). Twelve months later, mice were sacrificed, one lobe of lung was fixed, embedded in paraffin for H&E staining ^14^ ^15,16^. *Gprc5a*-KO /*Aldh2*-KO mice were generated by cross-breeding *Gprc5a*-KO mice with *Aldh2*-KO mice. Lung tumorigenesis and metastasis were compared among mice in four groups: i) wild-type (WT); ii) *Aldh2*-KO; iii) *Gprc5a*-KO; iv) *Aldh2*-KO/*Gprc5a*-KO.

**Cell lines and Cell Culture**

Primary mouse lung cancer cells (S1601) were obtained from *Gprc5a*-KO mouse lung tumor at the age of 14 months, and the cells were cultured with DMEM (Hyclone) supplemented with 10% FBS (Gibco). Non-small cell lung cancer cell (NSCLC) lines, A549, H1792 were as described as previously ^4^.

**Bioinformatic Analysis**

The comparison data of ALDH2 mRNA differential expression in paracancer tissues and tumor tissues of all types of cancer were searched and analyzed from TCGA database obtained from the Gene_DE module of TIMER2.0 website. Next, the TCGA database obtained from Kaplan-Meier Plotter was used to search and analyze the differences in disease-free survival and overall survival in lung adenocarcinoma between high and low expression of ALDH2 mRNA level. ALDH2 expression in patients with different pathological stages of lung adenocarcinoma was searched from the TCGA database obtained from the GEPIA website.

**Western-Blot**

To prepare whole-cell or tissue extracts, cells or tissues were lysed by RIPA lysis buffer (150 mM NaCl,1% Nonidet P-40, 0.1% SDS, 0.5% deoxycholate, 50 mM Tris (pH 8.0), 25 mM NaF, 2 mM Na3VO4, 5 mM PMSF and 2 mg/mL of aprotinin (0.2% v/v) containing 5% bovine serum albumin (BSA). Antibodies against ALDH2 (Abcam, ab108306) and β-actin (MBL, PM053-7) were used for the blots. The secondary antibody was goat anti-rabbit or anti-mouse horseradish peroxidase (HRP)-conjugated IgG. All antibodies were diluted according to manufacturers’ instructions. ECL kit (Millipore) was used to detect the protein bands.

**Transfections and Viral Infections**

HEK293T cells were transfected with lenti-shALDH2 (or nonspecific sequence) plasmid or lenti-Aldh2 (or GFP) plasmid to knock down Aldh2. Lipofectamine 2000 (plasmid: lipo2000 1:4) was used as transfection reagent. The medium was replaced after 4-6 h. After 48 h the lentivirus medium was collected to infect lung adenocarcinoma cells. The stably transfected cells were selected by puromycin (2 μg/ml) or blasticidin (10 μg/ml). Then, the cells were expanded and collected for subsequent analyses.

**Transwell Assay**

The S1601 and S1601-shAldh2 cells were seeded on the top well of the transwell chambers (Costar) at a density of 2x104 /well. A total of 1ml of the conditional medium was added in the bottom chamber. Following cultivation for 24 h, the top well surface of the filter was wiped with a cotton swab to remove the cells. Then the migrated cells were fixed with 4% paraformaldehyde for 20mins. Washed cells were stained with 0.1% crystal violet for 1 h. The migrated cells were observed and captured by an inverted microscope.

**H&E Staining**

Tissue paraffin slices were dewaxed using xylene and passed through an ethanol gradient to hydrate. The sections were immersed into hematoxylin for 3-8mins to stain the nucleus, washed with water, processed with 0.6% ammonia water, and rinsed with running water. Next, the slices were stained with Eosin for 1-3mins to stain cytoplasm. Lastly, the sections were dehydrated using gradient ethanol and xylene and sealed with neutral gum. Images were acquired and analyzed by an inverted microscope.

**Ethanol Treatment**

For acute ethanol exposure, WT, *Aldh2*-KO, *Gprc5a*-KO, *Gprc5a*-KO/*Aldh2*-KO mice were injected intraperitoneally (i.p.) with ethanol. The total dose of 5.8 g/kg body weight was split into two injections separated by 4h. Ethanol (96%, Sigma) was diluted to 28% v/v in saline, and administered twice as 13 ml/kg body weight.

**Immunofluorescence and Confocal Microscope**

Paraffin-embedded mouse lung sections were deparaffinized, hydrated and blocked to reduce nonspecific binding. Sections were stained for surfactant protein A (SPA) (1:50; sc-13977; Santa Cruz Biotechnology, Santa Cruz, CA) and CC10 (1:50; sc-9773; Santa Cruz Biotechnology, Santa Cruz, CA). Secondary antibody staining was performed with donkey anti- rabbit 488 (1:2000; A21206; molecular probe, Invitrogen, Carlsbad, CA) and donkey anti-goat 555 (1:2000; A21432; molecular probe，Invitrogen, Carlsbad, CA). The nuclei were counter-stained with DAPI (Sigma). Confocal microscopy was performed with a Nikon N1 and images were processed with cooled CCD camera and NIS Viewer software.

**Cell-Sorting and Analysis**

Mice at 12 weeks of age were sacrificed, and the lungs were perfused with 30 ml of PBS solution through the right ventricle until cleared of blood. The tracheas were injected with 1 ml dispase (354235, Corning Incorporated) and 1 ml 1% LMP agarose. The lungs were placed on ice, chopped into pieces, incubated with 0.001% DNase (D4527, Sigma) and 2 mg/ml of collagenase/dispase (10269638001, Roche) in PBS at 37℃ for 45 min. The tissue lysates were filtered through 100 μm and 40 μm pore-sized filters and centrifuged at 800 rpm for 5 min at 4^o^C. Cells were resuspended in red blood cell lysis buffer (R7757, Sigma) for 1 min, washed in PBS, resuspended in PBS/10% FBS (PF10) and incubated 15 min at 4^o^C with the appropriate antibody for surface marker staining. BASCs were isolated by sorting for the Sca-1^+^/CD45^-^/CD31^-^/CD34^+^ cell population, AT2 were isolated by sorting for the Sca-1^+^/CD45^-^/CD31^-^/CD34^-^ cell population; all cell populations were isolated using a FACS sorter (Becton Dickinson). Sca-1-FITC (557405), CD45.2-Biotin (553771), CD31-Biotin (558737), CD34-PE (551387), and Streptavidin-APC (554067) were from BD Pharmingen.

**Statistical Analysis**

Comparisons among groups were performed by the Student t test or Tukey-Kramer comparison test followed by analysis with GraphPad Prism Software (GraphPad Software). A P < 0.05 was considered significant (* P < 0.05; ** P < 0.01; *** P < 0.001; ****, P < 0.0001).

**Supplementary Data**

**Fig. S1** ALDH2 deficiency is associated with lung tumor progression. **(A)** mRNA expression of ALDH2 in multiple tumor tissues and adjacent normal tissues from the TIMER database. Lung adenocarcinoma (LUAD) and lung squamous cell carcinoma (LUSC) are marked in red. **(B)** ALDH2 mRNA expression in lung adenocarcinoma tissues with different pathological stages from the GEPIA dataset. ("Stage I" vs. "Stage IV", P = 0 .0172); ("Stage I" vs. "Stage III", P = 0.0377) **P* ＜0 .05. **(C)** Overall survival (%) curve in LUAD patients with low or high ALDH2. mRNA data were obtained from TCGA database. According to ALDH2 mRNA level, patients were grouped as high ALDH2 (n=123) and low ALDH2 (n=123). Log rank test P value is 0.0042.

**Fig. S2** ALDH2-deficiency enhances clonogenic activity of lung cancer cells. **(A, D)** Immunoblot of cell lysates from NSCLC cell lines, H1792, H1792-shALDH2, and A549, A549-shALDH2 transfectants. **(B)** Invasion of the cell lines in vitro. **(C, E, F)** Clonogenic assay in lung cancer cell lines, H1792, H1792-shALDH2, and A549, A549-shALDH2 transfectants (* *P* < 0.05).

**Fig. S3** ALDH2-deficiency enhances ROS and promotes migration of lung cancer cells. **(A, B)** ROS by flow cytometry in S1601, S1601-shAldh2, and A549, A549-shALDH2, treated with or without acetaldehyde (ACE) (1mM) for 24 h. **(C-D)** The migration of A549 and A549-shALDH2 cells, H1792 and H1792-shALDH2 cells that were treated with or without 1 mM ACE for up to 30h, was analyzed by a wound healing assay.

**Fig. S4** Aldh2 expression is repressed in tumor tissue in Gprc5a-KO mouse model of lung cancer. (**A**) Relative mRNA expression of Aldh2 in normal lung tissues in WT and *Gprc5a*-KO mice. (**B**) relative mRNA expression of Aldh2 in normal lungs (N) and lung tumors (T) derived from *Gprc5a*-KO mice (* *P* < 0.05).

**Fig. S5** BASCs are expanded in *Gprc5a*-KO and *Aldh2*-KO mice but suppressed in *Gprc5a*-KO/*Aldh2*-KO mice. **(A-B, D-E)** Immunofluorescent (IF) staining to detect cells at small and terminal bronchi (S/TB) that co-express SPA and CC10. The lung tissues were obtained from WT, *Gprc5a*-KO, *Aldh2*-KO, *Gprc5a*-KO/*Aldh2*-KO mice, with or without ethanol treatment for 48 hours. **(C, F)** Bar graph indicates percentages of terminal bronchi with the indicated numbers of BASCs. Bar=50 μm (A, C).

**Fig. S6** BASCs are expanded in *Gprc5a*-KO and *Aldh2*-KO mice but suppressed in *Gprc5a*-KO/*Aldh2*-KO mice. Analysis by flow cytometry of BASCs isolated from the lung of WT, *Gprc5a*-KO, *Aldh2*-KO, *Gprc5a*-KO/*Aldh2*-KO following treatment with ethanol, or saline. *P* < 0.05 (** *P* < 0.01; *** *P* < 0.001).

**Fig. S7** *Aldh2*-KO mouse lungs are intolerated to high level of ROS. **(A, C)** Schema of analysis of ROS and apoptotic cells by flow cytometry. (**B**) Analysis of ROS in lung cells from WT, *Gprc5a*-KO and *Aldh2*-KO mice treated with ethanol (EtOH) or without by flow cytometry. (**D**) Analysis of apoptotic cells from WT, *Gprc5a*-KO and *Aldh2*-KO mice treated with ethanol (EtOH) or without by flow cytometry via Annexin V staining.

**Fig. S8** ALDH2 deficiency plays a dual role in lung tumorigenesis and progression. Different roles of Aldh2-deficiency-induced ROS in lung tumorigenesis and tumor progression.
